# Comparative Pathology—Eczema

**Published:** 1888-07

**Authors:** William Alston Edgar

**Affiliations:** Professor of Veterinary Medicine, College of Agriculture, Downton, England


### Art. XVI.—COMPARATIVE PATHOLOGY—ECZEMA.

BY WM. ALSTON EDGAR, F.R.C.V.S.

*Professor of Veterinary Medicine, College of Agriculture, Downton, England.*

The tendency to study pathology in its widest sense regarding species of secondary importance appears to be steadily gaining ground, and this line of work must, from a scientific standpoint, be regarded as of even greater importance to the medical and veterinary professions than comparative anatomy and physiology.

It is most remarkable that as man has everything in common with the lower animals, from an anatomical and physiological point of view, that he should so long have been separated by the strong barrier of prejudice from his companions in disease and death, and have been assigned an imaginary place of isolation in the domain of pathology.

The veterinary profession must gladly welcome the ennobling of its work by this higher association, as hitherto disease in the domesticated animals has been chiefly studied in relation to its effects upon themselves and not its probable influence indirectly upon the health of mankind.

I must confess to being one of a number in our profession taking a moderate view of the transmissibility of disease from animals to man, and welcome comparative pathology for the universal benefit it must confer upon veterinary medicine, if strict comparison may be widely made in diseases usually looked upon as confined to one species.

For the most part, comparison has been made between those diseases which either directly or indirectly are communicable from man to the lower animals, and *vice versa.*

Mr. J. Bland Sutton's interesting work in comparative hypertrophies, malformations, etc., almost suggests common causes for pathological changes in all species. That such common cause exists in some panzootic maladies, such as glanders, anthrax, rabies, is clearly demonstrated. It is almost equally clear that in the group of communicable diseases between man and the lower animals a similar micro-organism may operate as a common cause, *e. g.*, tuberculosis and actinomycosis, although the organism may possess various points of difference and the pathological changes induced may become modified in different species. Again, a zymotic disease, such as variola, may be represented in several species, having many symptoms in common, and yet not be intercommunicable, *e. g.*, small-pox, cow-pox and sheep-pox, although it would appear there are some few persons who still believe that vaccination is small-pox modified by its transit through bovines.

Those, however, who hold this view do not attempt any explanation of the suspension of the natural law of reversion to original type, and show how it is that in a series of generations the virus does not regain its normal virulence in natural soil and again become capable of producing instead of protecting from small-pox.

Further, there are some diseases, such as diphtheritis and scarlet fever, possessing in man well marked and easily recognized symptoms, with a definite etiology, which diseases are *supposed* to have their counterpart in the lower animals. The former probably affects the gallinaceous family, but it requires far more demonstration before it can be accepted as affecting calves and pigs. I am strongly of opinion that the so-called cases of diphtheritis in the pig have been due to ingestion of anthrax blood or flesh which under certain circumstances, in the pig, produces malignant sore throat, tonsilitis and ulceration of pharynx and larynx, frequently causing death, although I am informed by an eminent bacteriologist that pigs possess complete immunity from anthrax, and this, he concludes, because the bacillus anthracis is not found in the blood and tissues after death.

All are probably familiar with the recent attempt to establish in the case of scarlet fever its identity in man and bovines, but with what success the issue best determines.

The work done, however, in this direction has had its negative value in establishing our ignorance in comparative pathology, and it is to be earnestly hoped in the interest of medical and veterinary students, that the proposed University of London may, in the event of its establishment, be enabled to found a chair of comparative pathology.

In America, comparative anatomy and pathology receive much more attention than in this country, and the JOURNAL OF COMPARATIVE MEDICINE AND SURGERY, published at Philadelphia, is superior to any corresponding periodical at the service of the profession in this country.

I am free to admit that a very large share of advantage in the study of disease of comparative lines must fall to the veterinary pathologist.

The splendid strides made during the last decade in medcine and surgery are capable in many directions of application in our profession. We can profit largely by applying general principles in dealing with disease, and by a study of current medical literature the veterinarian may trace analogy in etiology, semiology and treatment in a large proportion of diseases affecting the lower animals.

We have many advantages denied to practitioners of human medicine in following up our diagnosis and treatment by post-mortem examination, owing to the comparatively short lives of many of our patients, and thus by careful observation and a record of our work we may return to the sister profession at least a remunerative interest. For instance, in the subject of this paper—Eczema—the surgeon has few if any opportunities of demonstrating post-mortem observations in this disease, but to the veterinarian they present themselves frequently.

In the present state of our knowledge, however, one thing is especially clear, that any inquiry concerning diseases of the lower animals in their relation to public health, should be conducted conjointly by representatives of the medical and veterinary professions, as laboratory and clinical observations would then probably be more in harmony and calculated to secure the advancement of truth.

It is scarcely necessary that I should point out that the subject of this paper, Eczema, has no connection with the zymotic form, eczema epizootica, otherwise known as foot and mouth disease—my desire being to trace analogy in diseases other than of an infectious type.

Several diseases of great interest and common to different species might have been chosen, such as cancer, ovarian cysts, pneumonia, etc., but my selection of Eczema is due to a very considerable experience of that malady during the past eight years in most of the domesticated animals, together with the fact that comparatively little information is available for a study of it.

Although eczema is usually regarded and classified as a skin disease, I have some doubt if it is but exceptionally, *per se*, a cutaneous malady, but rather to be regarded as a subjective disease, an index whereby the pathologist may read the hidden pages of visceral derangement and structural change.

I am confirmed in this opinion by a large number of post-mortem examinations in the dog and pig, and in two in the horse, some of which I will subsequently quote.

Eczema may be defined as acute or chronic hyperæmia and inflammation of the derma, accompanied by, as its name implies, an exudation of serum from the cutaneous capillaries.

All the varieties of the disease described simply represent a series of pathological changes, and the terms usefully express the various objective signs of the malady, viz., redness, œdema, papulation, exudation, producing vesication, with rupture of vesicles and incrustation, subsequently followed by—in the more chronic forms of disease—desquamation of epidermis, thickening and induration (local or general), generally the former, frequently accompanied by fission or cracking of the skin. The subject of eczema is conscious of certain subjective sensations, such as intense burning, itching and pain; the two latter only can be demonstrated in the lower animals, although it may be reasonably concluded from manipulation of the diseased parts that the burning sensation is actually experienced; intense itching is patent in all animals ; pain is frequently evidenced upon palpation and by constitutional disturbance.

It is interesting to note the remarkable analogy in the varieties of eczema met with in man and domesticated animals ; almost every phase of the disease detailed by the late Sir E. Wilson may be compared, *e.g.*, I have observed :

*Eczema erythematosum*, or *rubrum* in the horse and dog.

*E. papulosum* in horse and dog.

*E. vesiculosum* in horse, cow, sheep, pig and dog.

*E. pustulosum* or *impetiginosum* in horse, pig and dog.

*E. ichorosum* in horse, cow and dog.

*E. crustaceum* in horse, cow and dog.

*E. siccum* in horse and dog.

*E. squamosum* in horse, cow (parasitic chemical, only), pig and dog.

*E. fissum* in horse and pig (Foot's case).

*E. hypertrophicum* in horse (extremities only).

The above varieties assume an acute or chronic form in the different species.

Acute eczema occurs in horses, cattle, sheep, pigs and dogs ; and chronic forms in horses, pigs (rare) and dogs only.

Again, correspondence in localization of the disease in the several species is remarkable. Local forms occur in man, horses, cattle, sheep and occasionally in dogs, *e. g.*, nasal and orbital eczema ; but in the pig and dog the disease in either variety is most commonly diffused ; labial and facial eczema in man, horse and sheep, the so-called *crusta labialis et facialis*, which represent *E. crustaceum* ; mammary eczema in cow, described by some writers as "blister-pox" and black-pox, which, however, frequently also exist on the soft skin from the umbilicus to the mammæ, but is commonly unnoticed, owing to its position. Orbital eczema is also seen in cow.

Local eczema in its chronic form is well represented in the extremities of man and horse in *E. fissum* and *hypertrophicum*, known to veterinary writers as *psoriasis carpi*

*et tarsi,* or, "mallenders" and "sallenders," and the chronic transverse fissures from knee to fetlock, described under psoriasis, and the indurations of "grease," sequelæ of eczema.

In the etiology of eczema, however, is found the most interesting and useful comparisons, as it appears fairly demonstrable that conditions which operate in its production in man are also conducive to similar results in the lower animals, modified in different species.

If this be true of eczema it may be equally so in many other pathological conditions, and it may be possible by such comparison to explain many obscure causes of disease in certain species, which, being occult in one, may be capable of demonstration in another, if, as appears more than probable, common causes have common results modified only by species and circumstances.

Causes may be divided in all animals into extrinsic and intrinsic.

In the human subject causes operating from without are referred to as irritants acting locally, *e.g.*, heat, moisture, friction, chemical agents, exposure to sun's rays, etc.

Similar causes are doubtless in operation in the lower animals with parallel results, but I think almost invariably associated with some constitutional disturbances acting as a predisposing cause.

For instance, it frequently happens that a flock of lambs or sheep are depastured upon vetches in the summer, and when a large quantity of thistles are present in the tares, a severe outbreak of labial and facial eczema may occur. This, however, happens more frequently when the lambs are receiving an additional diet of albumenoid food, as cake or peas. These outbreaks occur on other green feeding stuffs than vetches, but rarely when sheep are upon a green food diet only, although the pastures may be charged, as it were, with thistles.

Again, in the horse, flannel lined collars and pads are a frequent source of scapular and dorsal eczema to sensitive skinned animals, but the predisposing cause of the disease is invariably an important change of diet, or constitutional condition, affecting the excretory and digestive apparatus, the local manifestations being due to congestion and friction. I think exposure to continuous moisture operates as an entire cause in the production of eczema of the heels and pasterns in horses, which, in its later stages, may become transformed into the chronic condition described as grease. Also vesicular eczema of extremities due to irritation produced by *symbiotes spatheferens*, is, of course, due to external conditions only. I have seen several cases of eczema produced in cattle and sheep by chemical irritants—arsenic and paraffin oil—but must regard such cases as purely artificial conditions. I have never, to my knowledge, seen it produced by the action of the sun's rays, in any of the lower animals, their hair or wool covering probably preventing this.

Local mammary eczema in the cow, the blister-pox and black-pox (which is *E. crustaceum*, flies, dirt, etc.,) of milkers, is possibly caused by irritation from the hands in milking or lying in constant moisture, especially decomposing urine. I think the latter is the true exciting cause, as the eruption frequently exists on the soft skin from the udder to the sternum, although, as previously stated, it is frequently unnoticed in this position. I say possibly caused by irritation, etc., because I think this disease, manifested locally in the cow, is always primarily caused by constitutional conditions. A few weeks ago a severe case of orbital eczema occurred in a cow under my notice, the eruption being confined to the eyelids and sides of face; this was suppressed by systemic and local treatment; there was no explanation for its localization.

In the human subject causes constitutional are referred to under nervous shock, disturbed nervous functions affecting nutrition and circulation, in many cases caused by disordered digestion, painful dentition, menstruation, utero-gestation and parturition, eczema being not infrequently an indication of broken health. Many of the foregoing causes are in operation in the production of eczema in our patients, especially those referable to disturbed circulation and digestion.

It is difficult to define how far disturbed nervous function is a primary cause in any of the domesticated animals. Eczema is common in children during dentition. I have observed several outbreaks of the vesicular form in pigs during the eruption of the temporary teeth, but am not aware that this is a cause in any other species.

I have seen no cases in any animals which may be traced to œstrum, utero-gestation or parturition.

Two things have struck me as remarkable in the study of this subject, viz., the almost constant hepatic and occasional splenic changes in the lower animals, and in medical literature an almost total absence of any reference to either hepatic or splenic derangement as a complication of eczema, excepting so far as it may be inferred under dyspeptic conditions associated with hemorrhoids or varicose veins.

I am of opinion, from post-mortem examinations made in the horse, pig and dog, and from the line of treatment most successful in those animals, that the liver is the viscus which is, in a large proportion of cases, the primary cause of cutaneous disturbance. This, of course, will not apply to those cases of chronic eczema in the dog, which are coincident with cardiac disturbance or valvular disease with secondary hepatic changes.

Personally, I have been a subject of local eczema for many years, and it frequently occurs either in conjunction with or immediately after functional disturbance of liver.

Acute eczema is frequently fatal in very young pigs, *i. e*, from three weeks to six weeks old, and under the circumstances it is not infrequently reported for swine fever. A whole litter will sometimes die off and the only post-mortem changes remarkable are the intense congestion of the liver and the marked cutaneous eruption which is equally distributed over the whole skin surface down to the knees and hocks.

It may be of practical interest to make a few further remarks upon this disease in pigs. Although very fatal up to the age of six or seven weeks, it is rarely so after that time. It occasionally requires considerable care to differentiate between the vesicular and crustaceous variety of eczema and swine fever; the pathological changes being almost identical where the well marked cutaneous form of the latter disease is present.

In eczema, however, the temperature is not above 103.4, the *purple* condition of skin is not present and the pigs do not die; and it will also be noted that the attack is generally confined to the same litter, indicating an absence of infection, although several animals may be attacked.

It has struck me that heredity must play some part under such circumstances, as I have noted such cases where several dozens of animals have been exposed to the same dietetic conditions and sanitary surroundings.

In many post-mortem examinations of the dog I have noted the same marked congestion of liver as present in young pigs, but generally accompanied by considerable softening of structure. I may, however, explain that all, or nearly all, such observations have been made in dogs probably over three years of age, and subjects of recurrent eczema of many months or years' standing.

The mucous membrane of stomach and intestines is invariably normal. Several cases of cardiac disease, chiefly valvular, have been associated with hyperæmia and hypertrophy of liver accompanied by chronic eczema; with such lesions I have regarded the heart mischief as the primary cause of skin disease. The frequency of such cases in canine practice renders it important to make a most careful examination of the circulatory apparatus before venturing a prognosis.

I have no post-mortem evidence of liver disease in horses or cattle coincident with eczema, but I desire to draw attention to two cases in the horse of chronic localized vasicular eczema associated with diseased spleen.

Case No. 1, a brown horse, had been a subject of eczema of the extremities for two or three years, which required constant local applications to keep in check, also intermittent courses of medicine. No treatment, however, effected more than temporary relief. A few months before the animal was destroyed it suffered from an acute attack of labial eczema, involving also the inside of mouth, the vesicles breaking and healing readily. The animal, although generally a fairly good feeder, seldom looked well, and was dull at work, the lassitude being greater some times than at others. Before the final illness occurred the lassitude became so marked that the horse was rested. The appetite continued moderate and excretions normal, but there was marked pain upon percussion over the region of liver, arching of back, and grunting when turned suddenly round. The mucous membranes were always slightly icteroid. My diagnosis at this stage was enlargement of liver, but upon rectal examination I found that the spleen was enormously enlarged and several nodules about the size of walnuts could be felt through the bowel. The animal was destroyed and the post-mortem showed enormous splenic enlargement with a uniform diffusion of the tumors throughout whole structure of spleen, presenting upon section the characteristic features of lymphadenoma. I conclude, from a similar case at present under observation, that the eczema was caused by the condition of spleen.

In case No. 2 the animal is still living. It has been a subject of chronic facial eczema for two years or more. I am not aware that any treatment has been adopted; it came under my notice owing to staggering fits in the stable. I found cardiac disturbance, and consequently made an examination of liver and spleen. The latter was sufficiently enlarged to be felt *per rectum*, and I concluded that (owing to the animal being a white one) the disease was probably melanosis, although no external evidence of that condition existed.

While I refer to functional derangement and structural change of the liver as a very frequent cause of eczema in the lower animals, I am free to admit that many cases may be due to disturbance, from dietetic causes, of the digestive organs generally, and also renal changes, which, by constantly disturbing the vicarious cutaneous circulation, tend to establish the localized or general congestion which, with a naturally weakened skin tissue, is quickly followed by irritation and exudation.

In general practice by far the most frequent patients are horses and dogs. The latter are attacked in large numbers all the year round, probably owing to the artificial conditions under which so many dogs are compelled to exist.

I cannot trace any analogy in man and the lower animals in eczema as an indication of broken health, as a large percentage of cases occur in dogs and horses in a plethoric condition, especially the former, when being well fed and allowed but little exercise, the liver becomes inactive and eczema is commonly the result. That this is a fruitful source of the disease is evidenced by the salutary effect of diametrically opposite conditions being imposed when the animals are under treatment.

Eczema occurs occasionally in dogs after surgical operations and also as a complication during convalescence in some acute diseases, as distemper, and in the horse after influenza or any prolonged illness, when the animal is rapidly regaining strength and receiving large quantities of nitrogenous food.

Prognosis is identical in man, horse and dog; in each the disease is often tedious and difficult to cure, recurring at irregular intervals or seasons, patients being sometimes under treatment for many months, the ultimate issue largely depending upon the primary cause. I have seen no chronic eczema in sheep or cattle and only one case in the pig, a sow, in which the disease lasted for about four months and assumed the varieties of *E. vesiculosum*, *E. crustaceum* and *E. fissum*, well marked cicatrices being formed upon all parts of body. She was killed in a very fat condition with the disease in an active state.

Treatment by comparison is of special interest, and it may, I think, be fairly concluded that success lies in the direction of adoption of general principles, which may be generally followed in dealing with all species. Where causes are identical and the variety of diseases similar, treatment at the outset will be much the same in any animal. Of course, the selection of drugs will in many cases differ for simple convenience of exhibition or application. Idiosyncrasy, I feel convinced, plays an important part in domesticated animals as in man, for it may be often noted that certain drugs which act successfully in one animal will, when applied to another of the same species, under apparently precisely similar circumstances, have no curative effect.

Eczema, in one or more of its many varieties, is probably the most common skin disease by which the horse and dog is attacked ; dogs being, in my experience, affected with special frequency and in large numbers during the spring and summer. Chronic recurrent eczema is more common in this species than in any other.

Under almost all circumstances local and systemic treatment is necessary, the former, except in one or two chronic phases of local eczema in the horse, being rarely successful alone.

It is quite beyond the scope of a limited paper of this kind to enter into detailed treatment for the different varieties of the disease in all the species enumerated as affected by it, and I propose only to follow in this portion of my subject the same general line of comparison.

It is not always possible to define the exact cause or causes of each special attack in any given animal, but where a number are affected simultaneously the cause may be generally referred to dietetic arrangements, and under any circumstances a change of diet is beneficial if not essential. It is necessary to correct any dyspeptic conditions, if existing, and to promote by regular exercise hepatic and cutaneous circulation in order that the normal functions of the skin may be stimulated, and the tendency to localized congestion overcome. I think this essential exercise in the treatment of dogs and horses is a point often overlooked and omitted.

In the horse, where severe eczema of the heels exists, exercise cannot of course be had recourse to, or transversal cracking of the skin may possibly be caused.

In outbreaks of the disease in sheep and pigs an entire change of diet and situation in conjunction with the administration of salines for a couple of days is often the only treatment necessary. Liquor arsenicalis is with advantage given to sheep if any further treatment is called for ; but, as I have stated, these animals do not often suffer from a secondary attack or with the disease in a chronic form.

My further remarks will apply to horses, cattle and dogs.

A glance in Dr. Ringer's Therapeutics, down the list of drugs suggested in the treatment of eczema, will indicate at once that the action of medicines in man and the lower animals is identical, as one may choose from that list most of the general agents employed by veterinarians in the treatment of this disease.

I desire to call attention to two or three agents not generally used in the treatment of eczema, which may be employed with special advantage.

The most common varieties in the horse I find to be vesicular, pustular and perhaps dry eczema—*E. siccum*—where alopecia takes place all over the neck, shoulders and back. This is often a most obstinate form to treat. The catarrhal form, *E. ichorosum*, is not uncommon, generally acute on one large division of body, *e. g.*, the hind quarters or back. I have also seen some remarkable cases of this variety in cattle.

A mild saline purgative in the cow and an aloetic in the horse, where the animals are in good general condition, is the best preparatory treatment, followed for a few days by magnes. sulphate and potass. nitras in small doses. If the skin does not resume its healthy condition after the first effusion, a course of iron carbonate or liquor arsenicalis generally effects this result, although in some cases in the horse arsenic appears to be of questionable use.

Locally, no drug seems to act so well in arresting the catarrh and irritation as a solution of plumbi subacetas or hydrass. perchloridum, one part to one thousand in water. This is the least obstinate form of eczema in horses and cattle, although it generally attracts much attention, owing to the suddenness of attack and apparent severity.

Similar treatment in the vesicular form is pursued with advantage at the outset, but here there is a greater tendency to assume a chronic condition.

Under such circumstances the mucous membranes and liver must be carefully watched. Bi-carbonate of potash and soda replace the mag. sulph. and nitrate of potash, and a small dose of calomel is useful once a week or oftener —after a fortnight, liquor arsenicalis or bromide of arsenic alternated with a course of strychnine.

Externally,a stimulating lotion often relieves the intense itching, when lead lotions and ointments fail to do so. Here I may especially mention the remarkable effects in some cases resulting from the application of Professor Tuson's Sporokton, which, as is generally known, is a preparation of zinc and sulphurous anhydride, there being much free gas. This dressing certainly does act in some cases in the horse and dog almost as a specific. Its curative effects in "grease" as a result of eczema have only to be more fully recognized by the profession to be largely appreciated. Upon one occasion a lady who was suffering from eczema was so struck with its effect upon her dog, that she sent for a bottle for herself, and the result was equally satisfactory.

Where itching is intense and considerable surface has been denuded by the friction or scratching. the oleate of lead acts well.

*E. pustulosum* in the horse has given me more trouble than any other variety, and I am of opinion, from its recurrence year after year, that it is generally associated with hepatic changes ; under any line of treatment it is liable to recur. A frequent change of diet is useful.

A case came under my notice a few months since which had been under treatment for nearly a year by different veterinary surgeons. I suggested a liberal supply of mangold wurzel per day, and without any medical agents the animal was in a few weeks well. This recovery will, however, probably be only temporary. I have found little or no benefit from arsenic in some of these cases, but better results from hepatic stimulants.

This treatment, as indicated for vesicular eczema in the horse, is equally successful in the cow. The local mammary form is usually overcome by an unguent of diacetate of lead. In several large dairies in my practice, clients having a supply of this ointment, treat their cases of blister-pox without any constitutional agents except, possibly, a mild saline draught.

In the dog, *E. rubrum*, *vesiculosum* and *crustaceum* are the varieties met with in every day practice. Ten per cent. of such cases I think prove chronic and troublesome, some taxing one's knowledge of the pharmacopœia.

I think it a mistake to restrict dogs to one special form of diet, as is so frequently done. Animals, for instance, kept almost entirely in a house are allowed little or no flesh food, and a farinaceous diet entirely, pre-disposes dogs to liver congestion and eczema. Under such circumstances, an opposite diet is indicated ; this applies to any form of the disease.

At the outset, mild aperients, followed by hepatic stimulants and nerve tonics. I may here draw attention to the action of Euonymin. Having found from personal experiments that the drug was capable of producing an enormously increased excretion of bile, I was led to try it upon the dog in eczema, in conjunction with small doses of strychnia, and it acts, in very many cases, with remarkakle success.

The action of this drug has led me to conclude, in conjunction with post-mortem evidence, that liver congestion is a very frequent cause of eczema, quite apart from any disturbance of the stomach or intestines.

During the course of treatment, an occasional aperient is an advantage, also administration of potass. bicarb. in food. As tonic agents, quinine and strychnia, I think, act the best, given alternately with iron carbonate and sesquicarbonate of ammonia.

For local applications, solution of sub-acetate of lead in the acute form where exudation is great and the irritation intense, is, as in the horse, very useful, or oleate of lead or zinc benzole ointment, may be equally efficacious. When the skin has been much scratched, the ointment affords protection and comfort. The powders, useful in the human subject, are of little use in the lower animals, owing to the action of the panniculus muscle.

Where the disease has become more or less chronic and the skin tissue in a low nutritive condition, stimulants must be applied. I place foremost Sporokton—the strength of solution to be regulated by the condition of skin.

Tar, sulphur and turpentine ointment, freely applied, often stops the intense itching in the form *E. rubrum*, and in many cases where every other dressing has failed, iodide of sulphur ointment will effect a cure. Either of these agents also act well in the dry, diffused eczema of the horse, and in the local form affecting knees, hocks and back of legs, from centre to fetlocks. Ointment of belladonna extract and glycerine is very useful, softening down the skin and preventing the obstinate cracking and induration.

From this brief outline of the treatment adopted in eczema of the lower animals, it will be noted that certain medicinal agents give almost identical results, in all species from man downwards, which I think, in conjunction with the analogy demonstrated in Etiology and Semiology, tends to establish the advantage which must accrue, at any rate to veterinary medicine, from a study of comparative pathology.

The interest shown in this direction by medical workers, and the prominence given to the subject by the leading medical journals, should encourage our profession to share to its utmost ability, on this common battle-field, the burden of the fight with disease and death, the inheritance alike of man and his dumb companions and slaves.

